# Subnuclear Phacoemulsification to Reduce Corneal Injury in Nuclear Cataract Surgery: Evidence From a Randomized Controlled Trial

**DOI:** 10.1155/joph/1737599

**Published:** 2025-03-18

**Authors:** Han Wang, Rubing Liu, Rong Wang, Xuyang Wang, Furong Luo, Jifa Kuang, Zebin Li, Chengwu Yang, Mingbing Zeng

**Affiliations:** ^1^National State Key Laboratory, Zhongshan Ophthalmic Center, Sun Yat-Sen University, Guangzhou 510060, Guangdong, China; ^2^Hainan Eye Hospital and Key Laboratory of Ophthalmology, Zhongshan Ophthalmic Center, Sun Yat-Sen University, Haikou 570311, Hainan, China; ^3^Graduate Center for Vision Research, SUNY College of Optometry, New York 10036, New York, USA; ^4^Department of Ophthalmology, T. H. Chan School of Medicine, UMass Chan Medical School, Worcester 01655, Massachusetts, USA; ^5^Clinical Trials Office, Beth Israel Deaconess Medical Center, Harvard University, Boston 02115, Massachusetts, USA; ^6^Measurement and Outcome Section, Division of Biostatistics and Health Service Research, Department of Population and Quantitative Health Sciences, T. H. Chan School of Medicine, UMass Chan Medical School, Worcester 01605, Massachusetts, USA; ^7^Department of Obstetrics & Gynecology, T. H. Chan School of Medicine, UMass Chan Medical School, Worcester 01655, Massachusetts, USA

**Keywords:** cataract, corneal endothelial cells, randomized controlled trial (RCT), subnuclear phacoemulsification, surgical trial

## Abstract

**Objects:** To assess the safety and effectiveness of subnuclear phacoemulsification (SNP), a new technique for reducing corneal injury in nuclear cataract surgery.

**Methods:** This randomized controlled trial (RCT) was designed in March 2020 and carried it out from April 1 to September 30, 2020, including a 3 months' follow-up. We recruited 256 age-related hard nucleus cataract patients and randomly assigned them to two groups: the experimental group receiving SNP, and the control group receiving conventional phacoemulsification (CP). A single surgeon performed all the surgeries. We compared the two groups on the cumulative dissipated energy (CDE), phacoemulsification ultrasound time (UST), and complications for safety, as well as at multiple postsurgery follow-up timepoints on three major outcomes for effectiveness: visual acuity, central corneal thickness, and central corneal endothelial cell density.

**Results:** The two groups were well-matched in terms of demographics, nuclear density, and safety measures (ultrasound energy, phacoemulsification time, and complications). For effectiveness after surgery, compared to the CP group, the SNP group had better visual acuity and thinner central cornea postsurgery within 1 week and had higher central corneal endothelial cell density at the 1- and 3-month follow-up.

**Conclusions:** Compared to CP, SNP is more effective for reducing corneal injury in cataract surgery. The widespread application of this technology will greatly improve the safety of cataract surgery, especially hard cataract surgery.

**Trial Registration:** Chinese Clinical Trial Registry: ChiCTR2000031114

## 1. Introduction

Since Kelman's invention in 1967 [1], phacoemulsification cataract surgery has been the most important, efficient, and fastest surgical treatment for cataracts. Although the efficiency of phacoemulsification instruments has greatly improved after decades of development, damage of ultrasonic energy to the corneal endothelium and intraocular structure remains a primary complication [2]. These complications are especially concerning for hard nucleus cataracts [3, 4], where phacoemulsification requires a longer time and higher ultrasonic energy and is more likely to cause complications, such as postoperative corneal edema, loss of corneal endothelial cells, and even dysfunction of the corneal endothelium [5].

During surgery, a surgeon can significantly improve the operative efficiency by changing the parameter settings and using different emulsification techniques [6]. Since the invention of the divide-and-conquer cracking technique [7], various splitting techniques have been developed. Among these techniques, the splitting of cataracts into nuclear fragments of different sizes has become a prerequisite for successful phacoemulsification.

Currently, two theories explain the mechanism of corneal endothelial cell damage in phacoemulsification cataracts: heat injury theory and free radical injury theory [8, 9]. Many researchers suggest that the phacoemulsification tip should be as far away from the corneal endothelium as possible or that in situ phacoemulsification in the capsule is beneficial for reducing mechanical damage to the corneal endothelium [10]. Om Parkash used the “chopper shield” technique to protect corneal endothelium [11]. In this study, the chopper between the nucleus and corneal endothelium was beneficial for reducing the contact between the nucleus and corneal endothelium. However, it was not conducive to controlling the direction of the nucleus and adhering to the phacoemulsification tip, which reduced the efficiency of phacoemulsification. Li used an isolated capsulorhexis flap from femtosecond laser–assisted cataract surgery to protect the corneal endothelial cells [12]. However, most hospitals lack the necessary equipment or conditions for laser-assisted cataract surgery. In order to increase the safety of phacoemulsification surgery for hard nuclear cataracts and reduce damage to the corneal endothelium, new techniques for handling nuclear blocks during surgery are worth exploring.

To address this challenge through a close interdisciplinary collaboration, our team developed a new technique called subnuclear phacoemulsification (SNP). We hypothesize that our new SNP method is more effective in reducing corneal injury in hard nucleus cataract surgery than the current conventional phacoemulsification (CP) methods.

## 2. Methods

### 2.1. Trail Design

**Figure 1:**
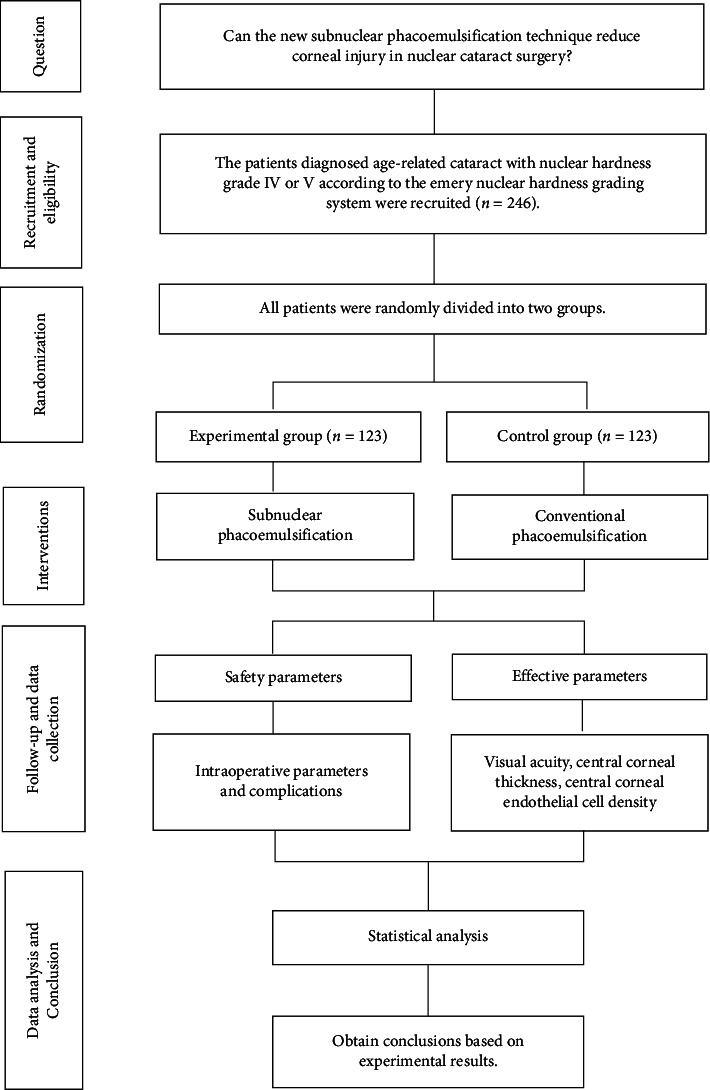
Profile picture of the randomized clinical trial. The picture shows the entire process of raising questions, patient recruitment, inclusion and exclusion criteria, random grouping, intervention measures, follow-up and data collection, data analysis, and conclusion acquisition in this clinical randomized controlled study.

This two-arm randomized controlled trial (RCT) involving 256 patients was conducted at a single site (Figure 1). In March 2020, three senior authors developed the study protocol, including but not limited to the study design, sample size estimation, randomization scheme, and statistical analysis plan. The study protocol was approved by the Institutional Ethics Committee (Institutional Review Committee [IRB]) (IRB # 2019001). We applied for grants and received funding from the National Natural Science Foundation of China (no: 82060176, PI: MZ) and the Natural Science Foundation of Hainan Province (no: 822MS190, PI: MZ). The study was conducted in accordance with the Declaration of Helsinki. Finally, we conducted this RCT at the Hainan Eye Hospital from April 1 to September 30, 2020.

### 2.2. Recruitment and Eligibility

We recruited all patients from a single clinical site. All study participants provided written informed consent, and their anonymity was maintained. The inclusion criteria were as follows: (1) 50–80 years of age, (2) diagnosed with age-related cataract, (3) nuclear hardness Grade IV or V according to the Emery nuclear hardness grading system, (4) intraoperative pupil dilation greater than 7 mm in diameter, and (5) corneal endothelial cell density greater than 1200 cells/mm^2^. Exclusion criteria were as follows: (1) chronic diseases such as hypertension, diabetes, renal insufficiency, and anemia; (2) other ocular diseases affecting eyesight, such as diabetic retinopathy, glaucoma, age-related macular disease, and uveitis; (3) history of internal ocular surgery; (4) corneal endothelial cell density lower than 1200 cells/mm^2^; (5) intraoperative complications such as posterior capsule rupture, Descemet's membrane detachment, hyphema, severe iris injury, and those whose corneal incisions require suturing, (6) unwilling or inability to follow-up after surgery. All patients underwent a standard preoperative examination after registration and provided written informed consent. Preoperative examinations included anterior segment analysis, central corneal thickness measurement, corneal endothelial cell counting, and intraocular lens power calculation.

### 2.3. Interventions

All patients underwent a standard preoperative examination after registration and provided written informed consent. The Topcon comprehensive optometer (Topcon, Tokyo, Japan) was used to assess the best-corrected logMAR visual acuity before and after surgery. We measured the central corneal thickness before and after surgery using Zeiss A-Oct (Carl Zeiss Meditec, Jena, Germany). The Topcon SP-1P with IMAGEnet Lite Version 4.25 corneal endothelium cell counter (Topcon, Tokyo, Japan) was used to measure the central corneal endothelial cell density. The Pentacam anterior segment analysis system (Pentacam, Münchholzhäuser, Germany) was used to measure the corneal curvature and astigmatism. The intraocular lens power was measured and calculated using a Zeiss IOL Master 700 (Carl Zeiss Meditec, Jena, Germany), and the Barrett Universal II formula was selected.

A single surgeon performed all operations in this study. With 25 years of professional experience, he has performed over 30,000 phacoemulsification and intraocular lens implantation procedures. The Infiniti Vision System was used in torsional continuous mode (Alcon, California, United States of America) for phacoemulsification with torsional amplitude 100%, linear ultrasound power 40%, vacuum 400 mmHg, and aspiration rate 35 cc/min. All surgeries followed a standard cataract phacoemulsification procedure, including capsulorhexis, hydrodissection, phacoemulsification, cortex aspiration, and intraocular lens implantation. A single-focus intraocular lens (PCB00, AMO, Illinois, United States of America) was implanted through a temporal 2.8 mm transparent corneal incision. The phaco-chop technique was used to crack the nucleus [13, 14].

**Figure 2:**
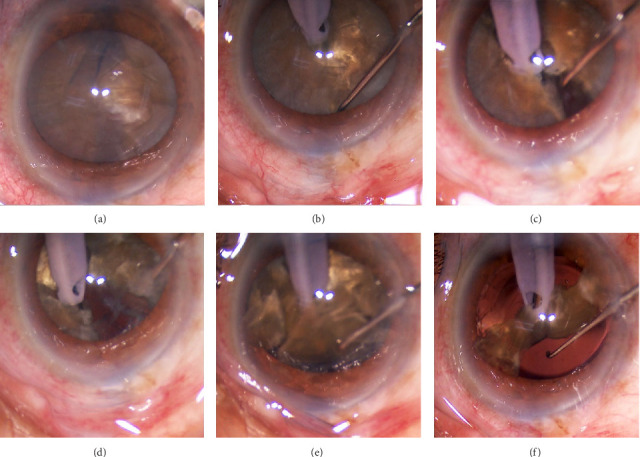
Intraoperative photographs show the operation process of subnuclear phacoemulsification. (a) Capsulorhexis after anterior capsule staining has been completed for hard nuclear cataracts. (b) Using the phaco-chop technique, the phacoemulsification tip is embedded in the center of the nuclear block, and the chopper hook reaches the equator of the nucleus to prepare for nuclear splitting. (c) The nuclear block is divided into two by centripetal longitudinal force. (d) The whole core block is divided into six to eight blocks. (e) Using the subnuclear phacoemulsification technique, the cracked block is turned over and covers the phacoemulsification tip. (f) When emulsifying the last one or two nuclear blocks, the intraocular lens is implanted first, and then SNP is used for phacoemulsification.

**Figure 3:**
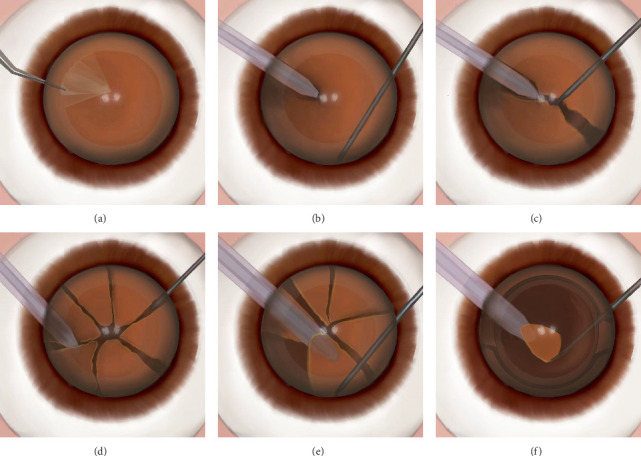
Drawn schematic photographs show the operation process of subnuclear phacoemulsification. (a) Capsulorhexis with forceps. (b) The phacoemulsification tip is embedded in the center of the nuclear block and the chopper hook reaches the equator of the nucleus. (c) The cataract nucleus is divided into two pieces. (d) The entire core block is divided into several blocks. (e) The cracked block is turned over and covers the phacoemulsification tip using the subnuclear phacoemulsification technique (SNP). (f) When the last one or two nuclear blocks are emulsified, an intraocular lens is implanted first, and then the SNP is used for phacoemulsification.

SNP, the experiment group: Due to the hard nucleus of cataract, it is best to split into six to eight blocks or more before phacoemulsification (Figures 2(a), 2(b), 2(c), and 2(d) and Figures 3(a), 3(b), 3(c), and 3(d)). In the experimental group, when emulsifying the nucleus, we turn the nuclear fragments over the phacoemulsification tip and cover the phacoemulsification tip to emulsify and remove the nucleus (Figures 2(e) and 3(e)). To prevent posterior capsule rupture, it is best to use Intraocular Lens Soft Shell Technology when the last one to two lens nuclei are emulsified and aspirated [15] (Figures 2(f) and 3(f)). Since the phacoemulsification is conducted under the nuclear block, we call it SNP technology.

**Figure 4:**
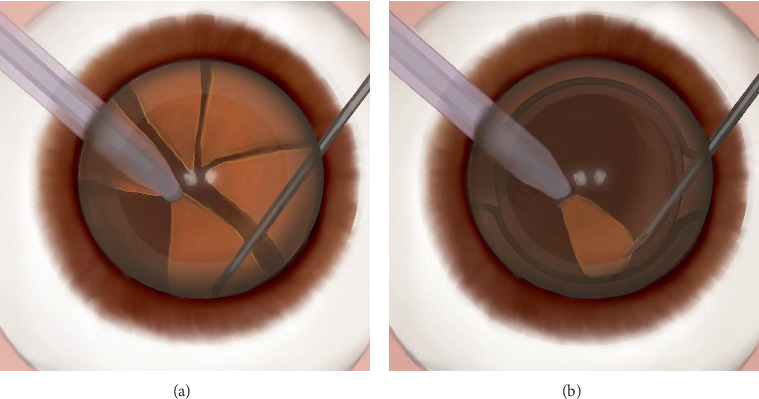
Drawn schematic photographs show the operation process of conventional phacoemulsification. (a) When capsulorhexis and nucleus splitting are complete, the nucleus block is pulled to the phacoemulsification tip and sucked to complete phacoemulsification. (b) When the last one or two nuclear blocks remain, the intraocular lens is placed first, and then the conventional method is used for phacoemulsification.

CP, the control group: In the control group, the split nuclear block was adsorbed on the phacoemulsification tip, and the phacoemulsification was conducted in situ in the capsule. There was no nuclear block in the anterior chamber. The operator could observe the whole process of the phacoemulsification tip and its phacoemulsification adsorption, and we call it CP (Figure 4(a)). When the last one or two nuclear blocks are left, the intraocular lens is placed first, and then the conventional method is used for phacoemulsification (Figure 4(b)).

After surgery, tobramycin/dexamethasone eye suspension 0.3%/0.05% (Toradex, Alcon Inc, Fort Worth, Texas, USA) and antibiotics (0.3% levofloxacin; Thornton, Osaka, Japan) were administered locally four times a day, and the dose was gradually decreased after 2 weeks.

### 2.4. Outcome Measures

#### 2.4.1. Safety Measures

##### 2.4.1.1. Intraoperative ultrasonic Energy

During surgery, the cumulative dissipated energy (CDE) of each case according to the readings displayed on the phacoemulsification machine (Infiniti, Alcon, California, United States of America) was recorded at two decimal places.

##### 2.4.1.2. Phacoemulsification Time

For each patient, we also recorded the ultrasound time (UST), in seconds, as their phacoemulsification time (Infiniti, Alcon, California, United States of America).

##### 2.4.1.3. Intraoperative Complications

We recorded the following possible intraoperative complications in each patient: posterior capsule rupture, incision burn, Descemet's membrane detachment, and iris injury.

#### 2.4.2. Effectiveness Measures

##### 2.4.2.1. Visual Acuity

On the first and seventh days, 1 month, and 3 months after surgery, the logMAR best-corrected visual acuity was measured and recorded. The range is −0.3–1.0, with smaller values indicating better visual acuity.

##### 2.4.2.2. Central Corneal Thickness

On the first and seventh days, 1 month and 3 months after surgery, the central corneal thickness was measured and recorded.

##### 2.4.2.3. Central Corneal Endothelial Cell Density

At 1 month and 3 months after surgery, the central corneal endothelial cell density was measured and recorded. A higher density suggests better effectiveness because it indicates less cell loss during surgery.

### 2.5. Sample Size

Since we measured the primary effectiveness outcomes several times after surgery and investigated the effects of the two factors, time and group (CP vs. SNP), we used a two-way multivariate analysis of variance (MANOVA) for our sample size estimation. A total sample size of 196 (98 per group, two groups) was needed, with the following parameters: (1) medium effect size of 0.25, (2) alpha level of 0.05, (3) power of 0.80, (4) 1:1 equal allocation of the two groups; and (5) five repeated measures of each patient. Assuming that 20% of patients will drop out during the planned 3-month follow-up, a total sample size of 246 (=196/0.8) was needed, 123 per group. We used the GPower software package Version 3.1.9.4 [16] (https://www.psychologie.hhu.de/arbeitsgruppen/allgemeine-psychologie-und-arbeitspsychologie/gpower) for sample size estimation.

### 2.6. Randomization

To ensure that the two groups were balanced, we used permuted block randomization (PBR). One senior author generated a randomization scheme using the SAS PROC PLAN [17]. Finally, 246 patients were randomly assigned to the two groups in equal proportions.

### 2.7. Blinding

Postoperative follow-ups and examinations were performed by inspectors blinded to the experiment. The data managers and statisticians were blinded to the assignment of the patients to the two groups.

### 2.8. Statistical Analysis

The IBM SPSS software package (IBM, Armonk, New York, United States of America, 2017) was used for all statistical analyses. Depending on the nature of the data, we applied analysis of variance (ANOVA), *t*-test, and chi-square test. For the primary outcomes, that is, the three effectiveness outcomes after surgery, we applied the MANOVA of repeated measures, as detailed in the sample size estimation section. We chose the conventional alpha level of 0.05 for statistical significance.

## 3. Results

All patients in the two groups were successfully followed up for 1 day, 7 days, and 1 month after surgery. Three months after the operation, there were five patients in the SNP group and four in the CP group. The data of these patients were excluded from the statistical analysis.

### 3.1. Demographics and Cataract Nuclei Hardness Grading

The two groups matched very well in terms of demographics (age, sex, and left/right eye) and cataract nuclei hardness (Table 1). None of the comparisons were statistically significant (*p* > 0.05).

### 3.2. Safety During Surgery

#### 3.2.1. Intraoperative CDE and UST and Complications

The SNP and CP groups did not differ in terms of the three safety measures (Table 2). In the SNP group, the mean CDE and UST were 18.29 ± 3.29 and 68.89 ± 5.56 s, and in the CP group, they were 18.57 ± 2.78 and 68.41 ± 5.94 s. None of the differences between the two groups was statistically significant (*p* > 0.05). Among the two groups of cases, one case in each group experienced posterior capsule rupture. No complications such as incision burns, Descemet's membrane detachment, or iris injury were found in either group.

#### 3.2.2. Effectiveness After Surgery

Table 3 summarizes the results of the comparison of the three effectiveness measures after surgery. Compared to the CP group, the SNP group had better visual acuity and a thinner central corneal within 1 month (as measured on the first and seventh days) and had higher central corneal endothelial cell density at the 1- and 3-month follow-up. The *p* values from the repeated-measures MANOVA were statistically significant (*p* < 0.05) for visual acuity and central corneal thickness. Although the difference between the two groups in corneal endothelial cell density was not statistically significant (*p*=0.140), the SNP group had a higher density in the first and third months. In the following, we explore the differences between the two groups for each of the three individual effectiveness measures.

#### 3.2.3. Postoperative Best-Corrected Visual Acuity

**Figure 5:**
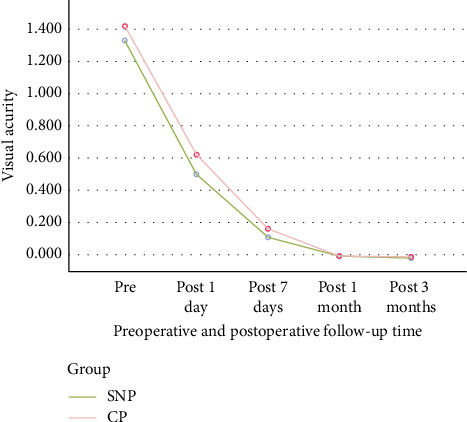
Comparison of best-corrected visual acuity. MANOVA for repeated measures of the three safety measures (Table 3) showed that the difference in best-recorrected visual acuity between the two groups was statistically significant. Repeated-measures ANOVA of the single safety measure of visual acuity also showed that this difference was statistically significant (*p* < 0.01). The following figure indicates that on the first and seventh days after surgery, the SNP group had better visual acuity than the CP group, and that this difference disappeared 1 and 3 months after the operation. SNP: subnuclear phacoemulsification; CP: conventional phacoemulsification.

The SNP group demonstrated better mean visual acuity compared to the CP group on the first and seventh days following surgery; however, this difference was resolved by the 1- and 3-month postoperative follow-up assessments (Table 3; Figure 5).

#### 3.2.4. Central Corneal Thickness

**Figure 6:**
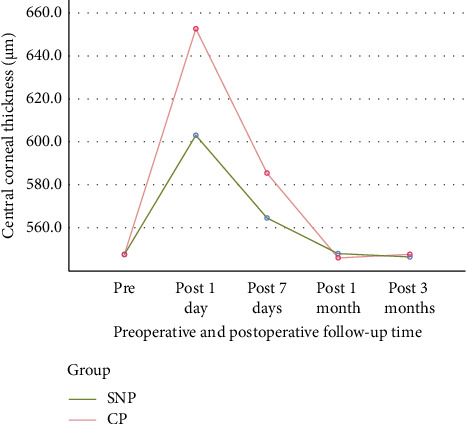
Comparison of central corneal thickness. MANOVA for repeated measures of the three safety measures (Table 3) showed that the difference in central corneal thickness between the two groups was statistically significant. Repeated-measures ANOVA of the single safety measure of central corneal thickness also showed that this difference was statistically significant (*p* < 0.01). The following figure indicates that on the first and seventh day after the operation, the SNP group had thinner central corneas than the CP group, and that this difference disappeared at 1 and 3 months after the operation. SNP: subnuclear phacoemulsification; CP: conventional phacoemulsification.

The SNP group exhibited a thinner mean central cornea compared to the CP group on the first and seventh postoperative days, but this difference was resolved by the first and third months of follow-up (Table 3; Figure 6).

#### 3.2.5. Corneal Endothelial Cell Density

**Figure 7:**
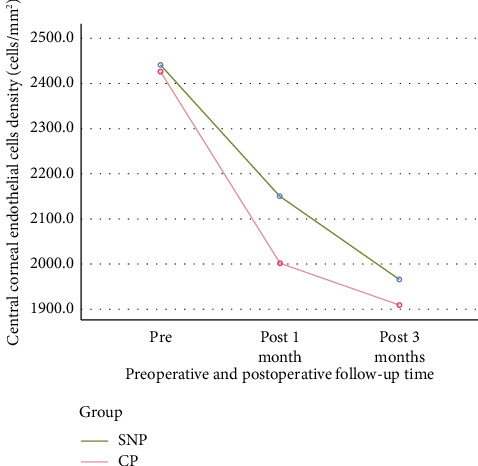
Comparison of corneal endothelial cell density in the central cornea. MANOVA for repeated measures of the three safety measures' results (Table 3) showed that the difference in corneal endothelial cell density in the central cornea between the two groups was not statistically significant; however, this can be caused by the fact that there were only three time points for this measure, while there were five time points for the other two measures. In addition, the SNP group tended to have a higher density in the first and third months. The ANOVA for repeated measures on the single safety measure of corneal endothelial cell density showed that this difference was statistically significant (*p* < 0.01). The following figure indicates that the first and third months after surgery, the SNP group had a higher corneal endothelial cell density than the CP group. SNP: subnuclear phacoemulsification; CP: conventional phacoemulsification.

On the first and third months after surgery, the SNP group had a higher corneal endothelial cell density than the CP group (Table 3; Figure 7).

## 4. Discussions

The principle of cataract phacoemulsification is to use the vibrations generated by high-frequency ultrasound to crush and absorb split nuclei. Ultrasound drives the phacoemulsification tip to cause high-frequency vibrations that can produce strong thermal effects and ultrasound waves. This can damage the corneal incision it contacts and even the intraocular structure and corneal endothelium far away from the phacoemulsification tip. Moreover, with an increase in the hardness of the nuclear block, more ultrasonic energy and time are required, and there is a greater chance of incision burns and corneal endothelial cell injury. Therefore, reducing the damage caused by ultrasonic energy to the intraocular structures, especially the corneal endothelium, has always been a goal pursued by medical device designers and clinicians.

The reason for the nonstatistically significant difference in corneal endothelial cell density through MANOVA was attributed to the lack of observations on the first and seventh days. While the other two effectiveness measures (visual acuity and central corneal thickness) had five repeated measures (pre-, first day, seventh day, first month, and third month), only three measures (pre-, first month, and third month) were recorded for corneal endothelial cell density. Some corneal endothelial cell counts could not be measured on the first and seventh days after surgery because of corneal edema. Thus, some statistical power was lost when a difference between the two groups was detected using this measure. Ideally, we would have measured this outcome at the same five time points as the two other measures, but difficulties prevented us from doing so. However, repeated-measures ANOVA of this single measure indicated that the difference between the two groups was statistically significant (*p* < 0.001, data not shown). Further studies with sufficient time points for this outcome are warranted to fulfill the power needed for MANOVA as per our study protocol.

Owing to the narrow space of the anterior chamber, the phacoemulsification tip easily touches the intraocular structure, especially when an anterior chamber surge occurs, leading to serious complications, such as posterior capsule rupture. Therefore, after completing the nuclear splitting, we suck the phacoemulsification tip into the nuclear block, pay attention to the specific process of nuclear block suction, and adjust the depth of the foot pedal to control the speed of the emulsion and avoid complications to the greatest extent [18, 19].

However, the exposed phacoemulsification tip can damage the corneal endothelium owing to high-frequency vibrations through the aqueous humor. If the nucleus can be placed between the phacoemulsification tip and the corneal endothelium, it will not affect the phacoemulsification or removal of the nucleus but will block the damage of high-frequency ultrasound and the heat generated by the vibration to the corneal endothelium.

Dense cataracts can be split into six to eight or more pieces before phacoemulsification. Before the last piece of the nucleus is emulsified, the other pieces of the nucleus can be overturned and covered on the phacoemulsification tip for nucleus emulsification, while the other pieces of the nucleus remain in the capsule, which can support and protect the posterior capsule and prevent its rupture. In soft nucleus cataracts, the nucleus is usually divided into four to six pieces. When the overturned nucleus was on the phacoemulsification tip and was quickly removed, its protective effect was not as prominent. One of the damage mechanisms of phacoemulsification of the corneal endothelium is that the large amount of perfusion fluid entering the anterior chamber has a mechanical flushing effect on the corneal endothelial cells. Using this technique, turning over the nuclei entering the anterior chamber is conducive to blocking the eddy current generated by the aqueous humor and reducing damage to the corneal endothelium.

During phacoemulsification, one of the mechanisms of corneal injury is the production of a large number of free radicals in the anterior chamber, resulting in damage to the corneal endothelium [8, 20]. The increase in ultrasonic energy and ultrasonic time was more prominent in hard nucleus cataract surgeries. However, using the new techniques provided in this study, it is possible to emulsify and remove almost all nuclear blocks above the phacoemulsification tip, which greatly blocks the damage of the corneal endothelium caused by the eddy current and heat effect generated by ultrasound. Owing to the free radicals, although this new technology has been applied in hard nucleus cataract surgery, it causes more serious damage to the corneal endothelium, which can cause corneal edema and delayed visual recovery on the first day after surgery; however, most patients' corneal edema can disappear within 1 week after surgery. Therefore, if combined with free radical scavengers during surgery, it will have a better corneal protection effect [21].

To use this technique more efficiently, before phacoemulsification, it is better to split the whole nucleus, which is more conducive to phacoemulsification into fragments, piece by piece. Compared to conventional techniques, because the phacoemulsification tip is covered by a nuclear block, the new technique increases the difficulty of the operation and theoretically increases the risk of posterior capsule rupture. For experienced cataract surgeons, the supporting role of the remaining nucleus can be fully utilized, which significantly reduces the risk of posterior capsule rupture. Advantageously, this technique was developed based on the phaco-chop technique. If a surgeon has mastered the phaco-chop technique, it will be easy for the surgeon to master this. Originally, when splitting the nucleus, the cracked nuclear fragments are turned and cover the phacoemulsification tip for emulsification; therefore, the learning curve will be very short.

For an underexperienced cataract surgeon, owing to the high demand for skills for splitting the hard nucleus and maintaining intraoperative anterior chamber stability, this technique is not recommended. Otherwise, the risk of rupture of the posterior capsule increases.

### 4.1. Limitations

The primary limitation of this trial was the great need for surgical skills, as detailed above. Similar to all surgical trials, the surgeon's skill plays a pivotal role in the success or failure of this new and effective procedure to reduce corneal injury in nuclear cataract surgery. Other potential limitations include strict requirements for blindness, accurate measurement of both safety and effectiveness outcomes, rigorously designed study protocols, strong statistical support from senior PhD statisticians from the very beginning of the study design until the end of the manuscript writing and publication, and close interdisciplinary collaboration among investigators from different, highly related fields (e.g., clinical ophthalmology, optometry, and biostatistics). Not every research team can offer all these resources to ensure highly reproducible results using this brand-new procedure, the SNP.

### 4.2. Generalizability

Similar to all RCTs, although with high internal validity from the rigorous design, due to the clearly defined inclusion and exclusion criteria, this trial has relatively limited external validity. Findings from this trial should not be generalized to patients who do not meet the inclusion and exclusion criteria. Future studies aiming to generalize the findings from this RCT on SNP to a real-world study (RWS) are warranted and are the next step in our planned team effort The senior statistician cocorresponding author, Dr. Yang, is an expert on the relationship between RCT and RWS, with publications specifically addressing the generalizability issue from RCT to RWS [22]. In addition, Yang is the originator of a new and powerful design, the [23, 24] complementary RCT and RWS for efficacy, effectiveness, or implementation design (CREID). We are confident that through the CREID design, by adding an RWS to this RCT, we will greatly improve the generalizability of the findings regarding this new procedure. Currently, we are developing a protocol for the RWS portion of our SNP within the CREID framework invented by Dr. Yang. In 2020, an earlier version of CREID won the Sole Sylvan Green Award at the SCT Annual Meeting [25].

### 4.3. Interpretation

With all the solid evidence from this rigorous and implemented RCT, we believe that if all procedures were fully followed, compared to CP, our new SNP would be a more effective technique for reducing corneal injury in cataract surgery while maintaining the same safety level. This new technique requires further promotion and wider application in the field. We welcome colleagues from across the world to contact us for collective efforts to offer more solid evidence of the effectiveness and safety of this new procedure, with the common goal of improving patient health.

## Figures and Tables

**Table 1:** Comparison of demographics and cataract nuclei hardness grading between the two groups.

Demographics	SNP group	CP group	*p* value
Patients/eyes (*n*)	123/123	123/123	—
Age (*y*, mean ± SD)	66.0 ± 19.7	66.6 ± 20.5	0.91
Males/females (*n*)	60/63	59/64	0.90
Right/left eye (*n*)	62/61	63/60	0.90

*Nuclear density*			
Grade IV (*n* (%))	112/91.1	110/89.4	
Grade V (*n* (%))	11/8.9	11/8.9	0.67

*Not*e: The *t*-test was used for age comparison and the chi-square test was used for sex and nuclear hardness comparison.

Abbreviations: CP, conventional phacoemulsification; SNP, subnuclear phacoemulsification.

**Table 2:** Comparison of safety measures between the two groups.

Outcome	Measure	SNP group	CP group	*p* value
Intraoperative ultrasonic energy	Cumulative dissipated energy (CDE, mean ± SD)	18.2 ± 3.2	18.6 ± 2.8	0.47

Phacoemulsification time	Ultrasonic time (UST, seconds, mean ± SD)	68.9 ± 5.6	68.41 ± 5.9	0.57

Intraoperative complications (# of events)		1	1	> 0.99
	Posterior capsule rupture	1	1	> 0.99
	Incision burn	0	0	> 0.99
	Descemet's membrane detachment	0	0	> 0.99
	Iris injury	0	0	> 0.99

**Table 3:** Comparison of effectiveness measures between the two groups at different timepoints after surgery.

Outcome	Group	Pre	1^st^ day	7^th^ day	1^st^ month	3^rd^ month	*p* value for group	*p* value for time
Visual acuity (LogMAR)	SNP	0.87 ± 0.17	0.46 ± 0.04	0.12 ± 0.02	0.06 ± 0.07	−0.02 ± 0.03	0.004	0.000
	CP	0.79 ± 0.30	0.70 ± 0.07	0.17 ± 0.07	0.01 ± 0.02	−0.04 ± 0.00		

Central corneal thickness	SNP	554.6 ± 15.6	603.65 ± 115.4	562.5 ± 128.3	555.2 ± 10.9	554.2 ± 13.8	0.002	0.725
	CP	553.3 ± 17.9	672.03 ± 112.2	575.3 ± 106.3	559.9 ± 11.9	556.5 ± 16.3		

Corneal endothelial cell density	SNP	2742.3 ± 505.1	NA	NA	2452.3 ± 370.3	2451.4 ± 450.1	0.140	0.000
	CP	2736.6 ± 348.9	NA	NA	2367.2 ± 301.8	2340.9 ± 300.8		

## Data Availability

The data supporting this study's findings are available from the corresponding author upon reasonable request. These data are not publicly accessible due to privacy and ethical restrictions governing participant confidentiality.
